# Targeting the Integrated Stress Response in Cancer Therapy

**DOI:** 10.3389/fphar.2021.747837

**Published:** 2021-09-24

**Authors:** Xiaobing Tian, Shengliang Zhang, Lanlan Zhou, Attila A. Seyhan, Liz Hernandez Borrero, Yiqun Zhang, Wafik S. El-Deiry

**Affiliations:** ^1^ Laboratory of Translational Oncology and Experimental Cancer Therapeutics, Warren Alpert Medical School, Brown University, Providence, RI, United States; ^2^ Department of Pathology and Laboratory Medicine, Warren Alpert Medical School, Brown University, Providence, RI, United States; ^3^ Joint Program in Cancer Biology, Lifespan Health System and Brown University, Providence, RI, United States; ^4^ Cancer Center at Brown University, Providence, RI, United States; ^5^ Hematology/Oncology Division, Department of Medicine, Lifespan Health System and Brown University, Providence, RI, United States

**Keywords:** integrated stress responses, ATF4, CHOP, apoptosis, cancer treatment

## Abstract

The integrated stress response (ISR) is an evolutionarily conserved intra-cellular signaling network which is activated in response to intrinsic and extrinsic stresses. Various stresses are sensed by four specialized kinases, PKR-like ER kinase (PERK), general control non-derepressible 2 (GCN2), double-stranded RNA-dependent protein kinase (PKR) and heme-regulated eIF2α kinase (HRI) that converge on phosphorylation of serine 51 of eIF2α. eIF2α phosphorylation causes a global reduction of protein synthesis and triggers the translation of specific mRNAs, including activating transcription factor 4 (ATF4). Although the ISR promotes cell survival and homeostasis, when stress is severe or prolonged the ISR signaling will shift to regulate cellular apoptosis. We review the ISR signaling pathway, regulation and importance in cancer therapy.

## Introduction

**FIGURE 1:**
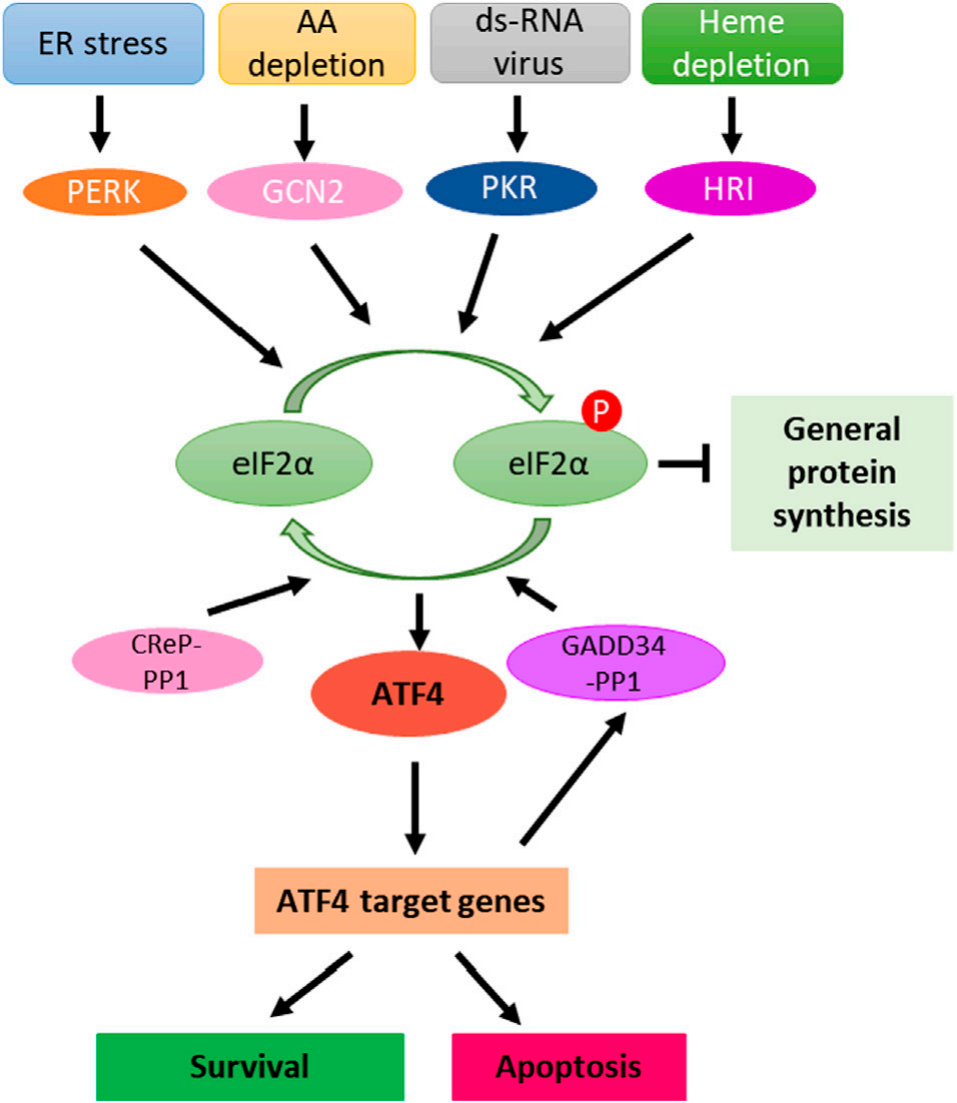
Integrated stress responses signaling pathway**.** ER stress, mitochondria stress or heme depletion, amino acid deficiency and ds-RNA virus infection activate PERK, HRI, GCN2 and PKR sensor kinases, leading to phosphorylation of eIF2α. eIF2α phosphorylation causes global inhibition of protein synthesis but selective translation of ATF4 mRNA. ATF4 binds to DNA targets to regulate the expression of genes that promote cellular adaptation, survival and apoptosis. Feedback regulation of ISR is regulated by constitutively expressed phosphatase complex CReP-PP1 and inducible phosphatase GADD34-PP1, which dephosphorylate eIF2α and attenuate or terminate ISR. AA, Amino acid; ER, Endoplasmic reticulum.

ISR is an evolutionarily conserved intra-cellular signal network activated in response to various intrinsic and extrinsic factors (Figure 1). Extrinsic factors include amino acid depletion, glucose deprivation, viral infection, hypoxia, heme deficiency, ROS (reactive oxygen species) and DNA damage (Pakos-Zebrucka et al., 2016; Clementi et al., 2020; Akman et al., 2021). Cellular intrinsic stresses, such as ER (endoplasmic reticulum) stress, can also activate the ISR (Pakos-Zebrucka et al., 2016). In the context of cancer biology, oncogene activation, such as MYC overexpression, can trigger the ISR (Tameire et al., 2019). Cancer cells with enhanced proliferation have enhanced protein synthesis which leads to a high basal level of the ISR as compared to normal cells (McConkey, 2017; Tameire et al., 2019). This may explain why ISR inducers can selectively target cancer cells.

Various stresses are sensed by four specialized kinases (PERK, GCN2, PKR and HRI) that converge on phosphorylation of serine 51 of eIF2α (Figure 1) (Perkins and Barber, 2004; Wek et al., 2006; Donnelly et al., 2013). Although significant sequence homology exists between these four eIF2α kinases in their kinase catalytic domains, underlying their common role in phosphorylating eIF2α, each eIF2α kinase possesses distinct regulatory domains and additional unique features that determine the regulation of these four kinases by signals that activate them (Donnelly et al., 2013). Each kinase responds to distinct environmental and physiological stresses, which reflects their unique regulatory mechanisms (Donnelly et al., 2013). eIF2α phosphorylation causes global reduction of protein synthesis and triggers the translation of specific mRNAs, including ATF4 to help with cell survival and recovery. However, if the stress cannot be reduced, ATF4 regulates an apoptosis program to eliminate the damaged cells (Pakos-Zebrucka et al., 2016; Costa-Mattioli and Walter, 2020).

ATF4 plays an important role in communicating pro-survival and pro-apoptotic signals. Once activated, ATF4 regulates transcriptional programs involved in cell survival (antioxidant response, amino acid biosynthesis and autophagy), senescence and apoptosis. The final outcome of ATF4 activation is dependent on the cell type, nature of stressors and duration of the stresses (Figure 1) (Wang et al., 2015; Wortel et al., 2017; Ojha et al., 2019; Tameire et al., 2019).

### The Integrated Stress Response and Cell Survival

**FIGURE 2:**
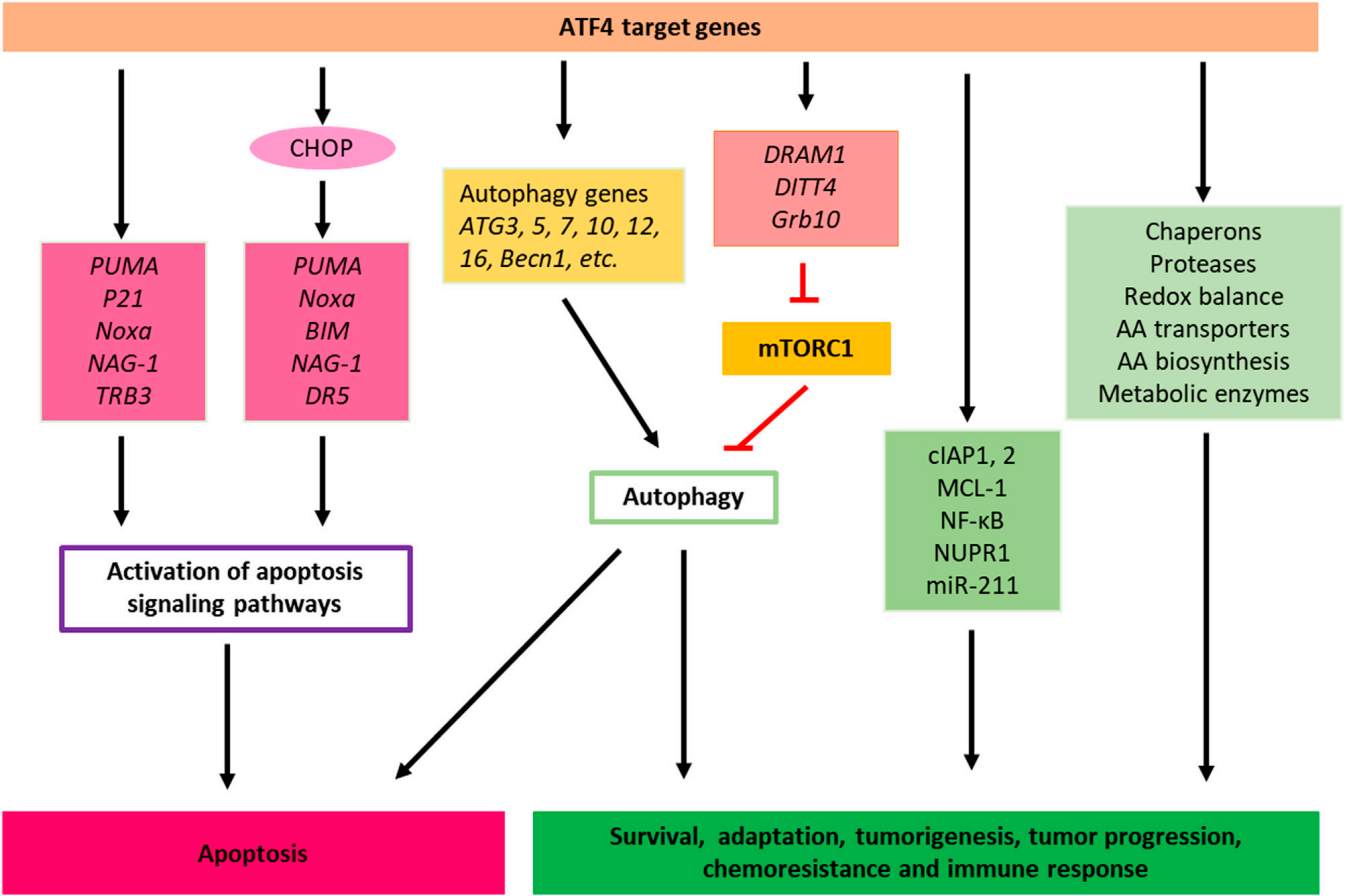
Cell death, pro-survival, tumor progression and chemoresistance pathways of ISR. ATF4 directly or indirectly controls the transcription of apoptotic, adaptive, tumor progression and chemoresistance genes. When stress persists (for example, drug treatments) and cancer cells are unable to adapt to and reach homeostasis though the activation of ISR, ATF4 shifts this balance towards apoptosis by inducing apoptotic genes. AA, Amino acid.

The ISR promotes cellular survival signaling by negative regulation of cell death pathways, such as apoptosis. For instance, as a consequence of ER stress, PERK‐induced activation of the ISR results in the expression of cIAP1 and cIAP2 (cellular inhibitor of apoptosis proteins) in tumor and non‐tumor cells (Hamanaka et al., 2009; Hu et al., 2004; Warnakulasuriyarachchi et al., 2004). Previously, it was demonstrated that restoration of the function of cIAP1 or cIAP2 in PERK^−/−^ murine embryonic fibroblasts during ER stress delays the early onset of ER stress-induced caspase activation and apoptosis seen in these cells (Figure 2) (Hamanaka et al., 2009).

ATF4 has also been demonstrated to facilitate anti-neoplastic agent bortezomib‐induced upregulation of anti‐apoptotic myeloid cell leukemia-1 (Mcl-1) protein, which is an anti-apoptotic Bcl-2 family protein that plays essential roles in multiple myeloma survival and drug resistance in many tumor types (Figure 2) (Hu et al., 2012).

It has been shown that both MCL‐1 and cIAPs can suppress apoptosis at different points in the apoptosis pathway that are upstream and downstream of the release of cytochrome c from the mitochondria. Mitochondrial cytochrome c plays a dual function in controlling both cellular energetic metabolism and apoptosis. It has been shown that, upon interacting with apoptotic protease activating factors (Apaf), cytochrome c can trigger the activation cascade of caspases once it is released from the mitochondria into the cytosol (Cai et al., 1998).

It has also been reported that miR‐211 is a pro-survival microRNA that regulates CHOP expression in a PERK‐dependent manner and thus PERK can mediate a pro‐survival function by suppressing a stress‐dependent expression of CHOP consequently leading to re‐establishment of cellular homeostasis before the initiation of apoptosis (Chitnis et al., 2012). In addition to its beneficial roles in restoring homeostasis, these ISR mechanisms may also contribute to tumor development. For example, an increased miR‐211 expression, found to be PERK-dependent, and was reported in mammary carcinoma and mouse models of human B‐cell lymphoma (Figure 2) (Chitnis et al., 2012).

Cancer cells use multiple stress response pathways such as the integrated stress response (ISR), cytosolic heat shock response (HSR), and unfolded protein response (UPR) mediated by organelles such as the endoplasmic reticulum (ER) and mitochondria to respond exogenous and endogenous or environmental stresses to evade apoptosis, ensure survival, proliferation, metastatic potential, and maintain cellular homoeostasis (OʼMalley et al., 2020). For example, to evade apoptosis and ensure survival, cancer cells may utilize the mitochondrial unfolded protein response (UPRmt) pathway and associated key proteins including chaperones HSP10, HSP60, and mtHSP70 and proteases ClpP and LONP1 to eliminate proteotoxic stress (Figure 2) (OʼMalley et al., 2020). Notably, upregulation of HSP60 expression and its upstream regulator ATF5 has been shown to enhance the apoptotic threshold in cancer cells resulting in therapeutic resistance in many cancer types. ATF-5 has been reported to regulate expression of Egr-1, BCL-2, and MCL1 to mediate proliferation and survival in cancer (Dluzen et al., 2011; Liu et al., 2011; Karpel-Massler et al., 2016).

Moreover, in addition to the genes mentioned above many other genes activated in response to ISR (Costa-Mattioli and Walter, 2020), including those encoding ATF4, ATF5 (Zhou et al., 2008); CHOP (C/EBP-homologous protein) (Palam et al., 2011); GADD34 (Growth Arrest And DNA-Damage-Inducible 34) (Lee et al., 2009); and in neurons, OPHN1 (Oligophrenin-1) (Di Prisco et al., 2014), other genes such as IBTKα (the α isoform of inhibitor of Bruton’s tyrosine kinase) (Baird et al., 2014) and NUPR1 (Nuclear protein-1), also play important roles in cell survival. NUPR1 has been found to play an important role in cell stress and stress-related apoptosis (Martin et al., 2021) and inactivation of NUPR1 promotes cell death by coupling ER-stress responses with necrosis (Santofimia-Castaño et al., 2018). More evidences suggest that ATF4 initiates the activity of transcription factor NUPR1. NUPR1 regulates the expression of several metabolic stress‐responsive genes, in particular, genes required in cell cycle regulation and DNA repair, as such, NUPR1 also is regarded as pro‐survival factors (Figure 2) (Jin et al., 2009; Hamidi et al., 2012).

Another gene activated during the ISR is the IBTKα which is activated during ER stress. IBTKα is a major substrate adaptor for protein ubiquitination and is an essential pro‐survival factor (Baird et al., 2014).

Likewise, eIF2α mediated translational repression has been suggested in activated B cell NF‐κB pathway induction as a mechanism to protect cells against ER stress (Deng et al., 2004). In a recent study, a pharmacologically activable version of PERK was used to uncouple eIF2α phosphorylation from stress and it was determined that eIF2α phosphorylation is both required and adequate to activate both NF‐κB DNA binding and an NF‐κB reporter gene (Deng et al., 2004). Also, HRI has been shown to be involved in NF‐κB activation (Abdel-Nour et al., 2019). This study found that the eIF2α kinase HRI controls NOD1 (Nucleotide-binding oligomerization domain-containing protein 1) signalosome folding and activation through a process requiring eIF2α, ATF4, and the heat shock protein HSPB8 (Abdel-Nour et al., 2019). Moreover, HRI/eIF2α signaling pathway was shown to be required for signaling downstream of the innate immune mediators including NOD2, MAVS (Mitochondrial antiviral-signaling protein), and TRIF (TIR-domain-containing adapter-inducing interferon-β) but dispensable for signaling pathways that rely on MyD88 (Myeloid differentiation primary response 88) or STING (Stimulator of interferon genes) (Figure 2) (Abdel-Nour et al., 2019).

### The Integrated Stress Response and Activation of Autophagy

Autophagy is a highly regulated eukaryotic cellular pathway that plays a major role in the lysosomal degradation of cytoplasmic unfolded proteins, peptides, damaged organelles or cytosolic components while also serving as a means to replenish depleted amino acids for building proteins and to provide energy to a starved cell. Autophagy can be activated by a variety of cellular stresses such as nutrient or growth factor deprivation, hypoxia, reactive oxygen species, DNA damage, protein aggregates, damaged organelles, or intracellular pathogens (Pakos-Zebrucka et al., 2016; Clementi et al., 2020; Akman et al., 2021). Autophagy can be activated both *via* specific, stimulus-dependent manner and more general, stimulus-independent signaling pathways to coordinate different phases of autophagy.

The ISR can modulate cell survival and cell death pathways through the activation of autophagy and the phosphorylation of eIF2α at S51 appears to be essential for stress‐induced autophagy (Pakos-Zebrucka et al., 2016). Autophagy can be integrated with other cellular stress responses through parallel stimulation of autophagy and other stress responses by specific stress stimuli, through dual regulation of autophagy and other stress responses by multifunctional stress signaling molecules, and/or through mutual control of autophagy and other stress responses.

#### PERK Regulates Autophagy

Although mechanisms by which phosphorylated eIF2α induces autophagy are still not completely elucidated, specific extrinsic and intrinsic stresses that lead to the phosphorylation of eIF2α have been demonstrated to trigger autophagy. For instance, ER stress increases phosphorylation of eIF2α and ensuing upregulation of certain autophagy receptors including *SQSTM1, NBR1*, and *BNIP3L* through PERK (Deegan et al., 2015). Likewise, inhibition of PERK pharmacologically suppresses transcriptional upregulation of these autophagy receptors in mammalian cells (Deegan et al., 2015).

Furthermore, phosphorylation of eIF2α mediated by PERK increases the conversion of ATG12 and LC3 due to the expression of polyQ72 aggregates in C2C5 cells, which is an essential step for autophagy formation (Kouroku et al., 2007). This PERK-mediated Unfolded Protein Response (UPR) has been shown to regulate autophagy from induction, to vesicle nucleation, phagophore elongation, and maturation (Deegan et al., 2013).

Moreover, it was reported that ER stress due to bluetongue virus infection of cells leads to autophagy through the activation of the PERK‐eIF2α pathway (Lv et al., 2015). The UPR which is initiated in response to the accumulation of misfolded proteins in the ER leading to stress is predominantly an adaptive response to the activation of the ISR. It was shown that UPR protects human tumor cells during hypoxia through regulation of the autophagy genes MAP1LC3B and ATG5 (Rouschop et al., 2010) and this was mediated by PERK phosphorylation of eIF2α. Conversely, abrogation of PERK signaling or expression of mutant eIF2α S51A which cannot be phosphorylated under the condition of hypoxia reduces the transcription of *MAP1LC3B* and *ATG5* (Rouschop et al., 2010).

IRS-induced autophagy also can lead to cell death. A recent paper reported that compound SH003 induces autophagy and autophagic cell death through a PERK-eIF2α-ATF4-CHOP signaling pathway in human gastric cancer cells (Figure 2) (Kim et al., 2020).

#### General Control Non-Derepressible 2 Regulates Autophagy

Similarly, amino acid deprivation in cancer cells leads to the phosphorylation of eIF2α mediated by GCN2 which is required for the activation of autophagy (Ye et al., 2010). Notably, while *GCN2* knockout cells exhibited decreased LC3 expression, cells with mutant the eIF2α S51A were not able to activate the processing of LC3 (Ye et al., 2010). Likewise, in the regulation of autophagy induced by amino acid starvation, phosphorylation of eIF2α at S51 was found to be required in yeast and mouse embryonic fibroblasts (MEFs) (Tallóczy et al., 2002). These findings suggest that eIF2α phosphorylation at S51 forms the central hub between different stresses and activation of autophagy.

Downstream of eIF2α phosphorylation, although ATF4 has been implicated to be essential for activation of autophagy, other mechanisms directed from eIF2α phosphorylation other than selective translation of ATF4 mRNA might also be involved in the activation of the autophagy process (Kroemer et al., 2010). It was previously suggested that phosphorylation of eIF2α might affect the ER in a manner that promotes the physical formation of the isolation membrane. Alternatively, eIF2α phosphorylation might stimulate autophagy through its effects on the transactivation of autophagy genes. eIF2α phosphorylation stimulates the selective translation of the ATF4 transcription factor, which stimulates LC3 expression which is essential for sustained autophagy (Milani et al., 2009; Kroemer et al., 2010). Furthermore, although autophagy interaction network components play important roles in vesicle trafficking, protein or lipid phosphorylation and protein ubiquitination and there are direct interactions between eIF2α subunits and core autophagy proteins, whether these interactions are biologically significant is not clearly understood (Behrends et al., 2010).

Under conditions of ER stress or amino acid deprivation, there is transcriptional upregulation of key autophagy genes mediated by ATF4 including *MAP1LC3B* and *ATG5* which are required for autophagosome biogenesis and function (Deegan et al., 2015; Rzymski et al., 2010; BʼChir et al., 2013)*.* ATF4 can also upregulate the DITT4/REDD1 and DRAM1, which represses the activity of mTORC1, subsequently inducing autophagy (Figure 2) (Kazemi et al., 2007; Whitney et al., 2009; Dennis et al., 2013; Tian et al., 2021).

Furthermore, ATF4 activation in response to amino acid deprivation also directs an autophagy gene transcriptional program by upregulating several autophagy genes such as *Atg3, Atg5, Atg7, Atg10, Atg12, Atg16, Becn1, Gabarap, Gabarapl2, Map1lc3b,* and *Sqstm*1 (Figure 2) (BʼChir et al., 2013)*.* Through the stimulation of key genes involved in autophagy, the ISR mediates the up-regulation of the autophagic process in an attempt to resolve the stress induced by amino acid deprivation. This is accomplished by the increased recycling of cytoplasmic components and sustaining the biosynthetic capacity of the cell and cellular ATP concentrations. The increased autophagic function leads to increased amino acid levels in ER required for *de novo* protein biosynthesis and similarly leads to increased levels of substrates including free fatty acids and amino acids for the tricarboxylic acid cycle (Rzymski et al., 2009; Ye et al., 2010).

However, it was also shown that a variety of autophagy genes can have a varying degree of reliance on ATF4 and CHOP signaling and that the transcriptional upregulation of such genes is regulated by the ratio of ATF4 and CHOP proteins that are bound to a particular promoter, and thus fine-tuning the expression of autophagy genes depending on the needs of the cell (BʼChir et al., 2013).

Studies on the effect of proteasome inhibition on survival signaling by the ISR have revealed that suppression of proteasome function pharmacologically using antineoplastic agent bortezomib results in depletion of amino acids in the ER required for protein synthesis leading to the activation of the ISR *via* GCN2 stress sensor (Suraweera et al., 2012).

Amino acid depletion as a result of proteasome inhibition also activates autophagy through mTOR in an attempt to restore amino acid homeostasis (Suraweera et al., 2012). Conversely, exogenous supplementation of essential amino acids depleted by the inhibition of proteasome function inhibition attenuates the phosphorylation of eIF2α and down-regulates autophagy (Suraweera et al., 2012). As such, depletion of amino acids by proteasome inhibition establishes a link between ISR activation and induction of autophagy in an attempt to sustain the survival of the cell.

#### Heme-Regulated eIF2α Kinase Regulates Autophagy

Although the other eIF2α kinases are present across different tissues, eIF2α kinase HRI is more specific to erythroid cells and plays a major role in erythrocyte differentiation during erythropoiesis (Suraweera et al., 2012). eIF2α kinase HRI mediates the translation of globin mRNAs with the availability of heme for the production of hemoglobin. By doing so, HRI protects erythroid cells from the increase of toxic globin aggregates under conditions of iron deficiency (Bruns and London, 1965; Chefalo et al., 1998; Han et al., 2001; Suragani et al., 2012). Other stresses such as arsenite-induced oxidative stress, heat shock, osmotic stress, 26S proteasome inhibition, and nitric oxide also were shown to activate HRI (Han et al., 2001; Lu et al., 2001; McEwen et al., 2005; Yerlikaya et al., 2008; Ill-Raga et al., 2015) and activation of HRI by these stresses is independent of heme and heat shock proteins HSP90 and HSP70 facilitates this process; however, the exact mechanism of HRI activation is still being studied (Lu et al., 2001).

A recent report demonstrated that HRI controls autophagy to clear cytosolic protein aggregates (Mukherjee et al., 2021). In that study, researchers found that the eIF2α kinase HRI induced a cytosolic unfolded protein response to prevent aggregation of innate immune signalosomes. Furthermore, they demonstrated that HRI controls autophagy to clear cytosolic protein aggregates when the ubiquitin-proteasome system is inhibited (Mukherjee et al., 2021).

Growth factor receptor-bound protein 10 (Grb10) is regulated by ATF4 (Zhang et al., 2018). the HRI-eIF2αP-ATF4 pathway suppresses mTORC1 signaling through Grb10 specifically in the erythroid lineage (Figure 2) (Zhang et al., 2018). mTORC1 was shown to act as a master regulator of autophagy since inhibition of mTORC1 was required to initiate the autophagy process (Dossou and Basu, 2019). It was also shown that mTORC1 directly regulates the downstream steps of the autophagy process, such as the nucleation, autophagosome elongation, autophagosome maturation and termination (Dossou and Basu, 2019).

#### PKR Regulates Autophagy

Talloczy, Z. et al. report that PKR acts as a potent inducer of autophagy during viral infection (Tallóczy et al., 2006). Also, two papers indicate that PKR is very important for the autophagic degradation of herpes simplex virions both *in vitro* and *in vivo* (Tallóczy et al., 2006; Orvedahl et al., 2007). In these settings, PKR was shown to operate upstream of Beclin 1 (Tallóczy et al., 2006).

**FIGURE 3:**
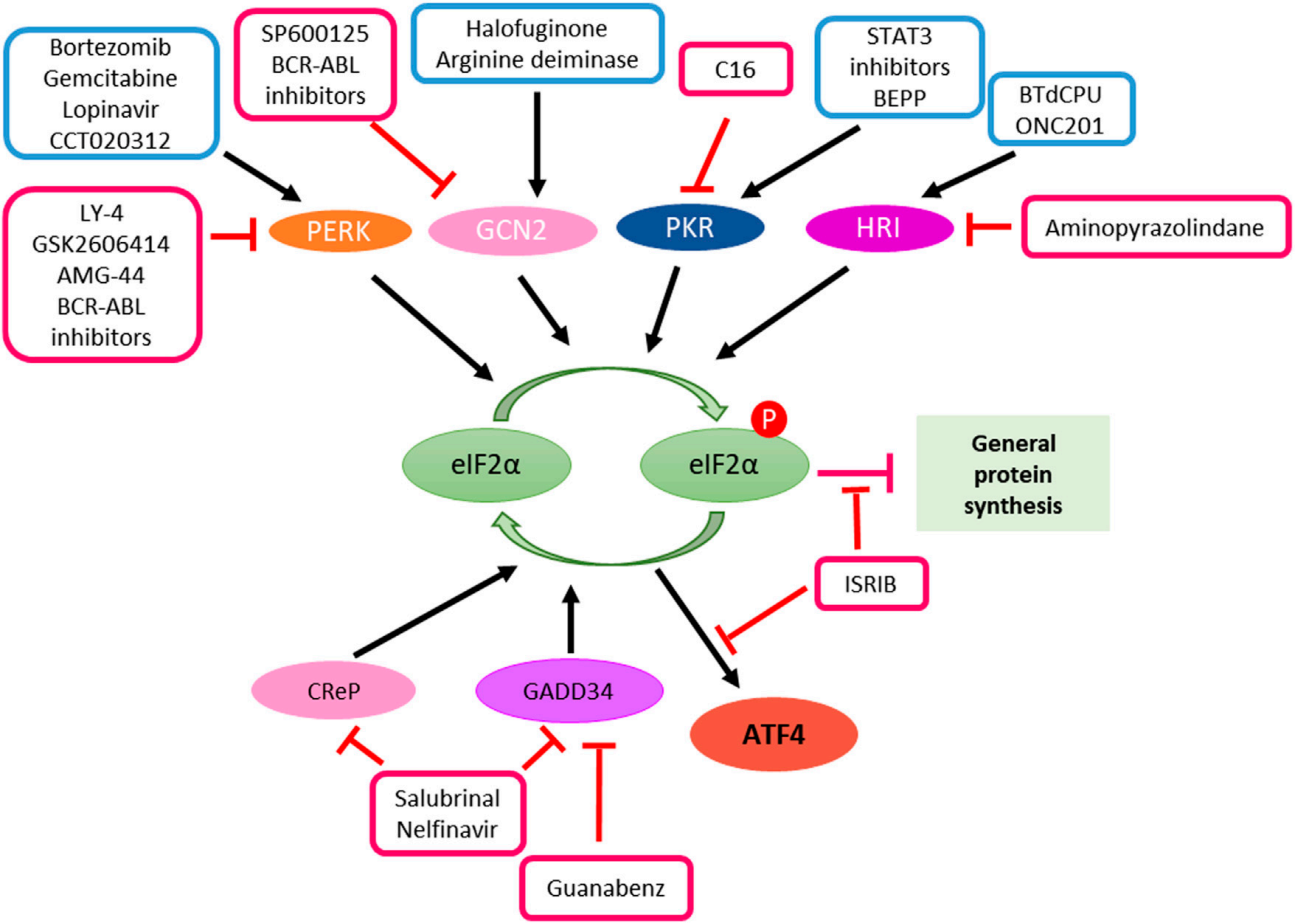
Manipulation of ISR in cancer therapy. ATF4 induction can be achieved either through kinase activators such as bortezomib, gemcitabline, lopinavir, CCT020312, halofuginone, arginine deiminase, STAT3 inhibitors, BEPP, BTdCPU and ONC201 or the inhibitors of phosphatases such as salubrinal, guanabenz and nelfinavir. In the case of ISR promotes cancer cell survival and resistant to therapeutic treatments, inhibition of ATF4 can be achieved by kinase inhibitors such as LY-4, GSK2606414, AMG-44, BCR-ABL inhibitors, SP600125, C16 and aminopyranzolindane or compound ISRIB downstream of eIF2α phosphorylation.

Shen, S. et al. report that STAT3 inhibitors (JSI-124, WP1066 and Stattic) caused the disruption of inhibitory STAT3-PKR interactions in human osteosarcoma U2OS cells, resulting in release and activation of PKR. PKR phosphorylates eIF2α, which regulates the activity of Beclin 1/Vps34 complex and facilitates autophagy induction (Figure 3) (Shen et al., 2012).

Pathogenic bacterium *Mycobacterium tuberculosis* (Mtb) infection induces the activation of PKR and PKR-mediated autophagy in macrophage. Sustained expression and activation of PKR reduced the intracellular survival of Mtb, which could be enhanced by Interferon gamma (IFNγ) treatment (Smyth et al., 2020).

### The Integrated Stress Response and Cell Death

The cell death pathways are complex and can be exploited by cancer therapeutic agents (Carneiro and El-Deiry, 2020). When stress persists and cells are unable to reach homeostasis despite the activation of stress response pathways, ATF4 can induce the transcriptional activation of apoptotic genes encoding CHOP (DDIT3) (Harding et al., 2000), TRB3 (Tribbles homolog 3) (Ohoka et al., 2005), and pro-apoptotic BH3-only proteins including PUMA (p53 upregulated modulator of apoptosis), Noxa (Phorbol-12-myristate-13-acetate-induced protein 1) and BIM (Bcl-2 Interacting mediator of cell death), thus leading to cell death (Galehdar et al., 2010; Altman et al., 2009; Puthalakath et al., 2007). ATF4 has been shown to regulate Noxa at the transcriptional level and this leads to the induction of apoptosis (Sharma et al., 2018; Núñez-Vázquez et al., 2021). Overall, through the induction of ATF4, this transcription factor appears to mainly trigger the intrinsic apoptosis by modulating the expression of pro- and anti-apoptotic BCL-2 family members. Interestingly, in the case of CHOP activation, induction of DR5 (Death receptor 5) mediated apoptosis appeared to be DR5 ligand binding independent and involving the engagement of FADD (Fas-associated protein with death domain) and caspase-8 (Figure 2) (Lu et al., 2014; Li et al., 2015).

Additional stresses such as those resulting from decreased mitochondrial translation (Sasaki et al., 2020) as well as the generation of reactive oxygen species (Kasai et al., 2019) have been shown to induce ATF4 expression. In the case of sustained mitochondrial deficiency, ATF4 response has been reported to lead to p53-mediated apoptosis (Evstafieva et al., 2014). Reactive oxygen species generated by Fenretinide treatment in neuroblastoma cells activates ATF4 leading to the induction of Noxa ultimately leading to apoptosis (Nguyen et al., 2019). In multiple myeloma cells, sensitivity to bortezomib treatment was associated with higher expression of ATF4 and loss of its expression lead to lower levels of Noxa, CHOP and DR5 (Narita et al., 2015). Recent work from our lab has also implicated ATF4 responsible for the induction of p53-target genes PUMA, Noxa, NAG-1(Nonsteroidal anti-inflammatory drug-activated gene-1)and DR5 upon treatment with prodigiosin analogue PG3-Oc (Figure 2) (Tian et al., 2021).

The aforementioned studies involve the induction of the ISR machinery in addition to distinct components of autophagy, cell cycle, and/or apoptosis pathway. This reflects the complexity of the interplay of these cellular pathways which remains underscored and likely to be context-dependent. Recent work has focused on post-translational modifications of ATF4 and how these affect the transcriptional control and cellular response. ATF4 has numerous sites that can be post-translationally modified including phosphorylation at various threonine and serine sites, methylation at arginine 239, and ubiquitination and acetylation at lysine residues (Wortel et al., 2017). These post-translational modifications affect ATF4 protein stability, activation and interaction with other proteins. In the case of apoptosis, methylation at arginine 239 by methyl transferase PRMT1 was found to be associated with the transcription of genes related to apoptosis (Yuniati et al., 2016). Further insight into ATF4 activation may shed light on understanding the context of how these transcription factors respond to stress and the biological outcome they ultimately trigger in both normal and cancer cells. Importantly, this will aid the intervention of novel therapies, the use of the ISR as potential biomarker for predicting therapy response and the combination of therapies that induce ATF4-mediated apoptosis. An example of therapy combination has been observed in *in vivo* neuroblastoma preclinical models with the BCL-2 inhibitor Venetoclax and Fenretinide (Nguyen et al., 2019). This studied combination highlighted the use of BCL-2 expression as a biomarker for neuroblastoma patients. A separate study in multiple myeloma suggested the use of ATF4 as a predictive therapy response biomarker for bortezomib and dexamethasone combination treatment (Narita et al., 2015). These studies exemplified the clinical translational applicability of exploiting the ISR in cancer therapy and highlight its warrant understanding to predict cancer types that will benefit from ISR modulating therapies.

### Dual Roles of the Integrated Stress Response in Cancer

The ISR plays different roles in tumorigenesis and tumor progression in different types of tumors. Hypoxia is a common phenomenon in solid tumors. It may induce apoptosis of tumor cells or tumor cells may develop the ability to adapt to the hypoxia or anoxic environment. Hypoxia can induce ISR gene expression in transformed mouse embryonic fibroblasts and the activated ER stress response confers resistance to apoptosis induced by hypoxia and thus facilitates tumor growth (Ameri et al., 2004). ISR mediator ATF4 is induced by anoxia in breast cancer cell lines (Ameri et al., 2004). The activated ISR plays an essential role in the adaptation to hypoxic stress allowing tumor cell survival under stress and is associated with resistance to therapy (Blais et al., 2004; Rouschop et al., 2013).

It was found that loss of extracellular matrix (ECM) attachment stimulates ISR signaling *in vitro*. And the activation of ISR further plays a critical role in resistance to anoikis and is required for metastasis (Dey et al., 2015). The ISR also has impact on the tumor microenvironment. Tumor cells undergoing ER stress can transmit ER stress to myeloid cells contributing to a pro-inflammatory tumor microenvironment, thus facilitating tumor progression (Mahadevan et al., 2011).

The role of ISR may be complex in tumors. In medulloblastoma, the ISR is activated, and the decreased ISR *via* gene manipulation attenuates medulloblastoma formation. Moderately enhanced ISR by gene manipulation noticeably increased the incidence of medulloblastoma, whereas a strongly enhanced ISR significantly decreased the incidence of medulloblastoma *in vivo*. Thus, the ISR plays dual roles in medulloblastoma formation (Stone et al., 2016).

Activation of the ISR is correlated with resistance to chemotherapy in pancreatic cancer and BRAF-mutated melanoma. Gemcitabine can induce ISR and the antiapoptotic pro-survival factors *via* the ISR pathway in pancreatic cancer cell line and the combination of gemcitabine + ISRIB which inhibits ISR induce more apoptosis *in vivo* (Palam et al., 2015). In BRAF-mutated melanoma, chronic ER stress involving induction of the ISR signaling pathway activates autophagy which contributes chemoresistance (Corazzari et al., 2015).

Triggering ISR can be a therapeutic strategy against cancer, since the ISR can induce apoptosis. ONC201 kills solid tumors by triggering ISR-dependent ATF4 activation and activation of the TRAIL-DR5 apoptotic pathway (Kline et al., 2016). In breast cancer, GBM and DMG cell lines, ONC201 induces ISR, TRAIL-DR5 and ultimately apoptosis (Zhang et al., 2021). The apoptosis increases with the enhancement of ISR induction by tazemetostat. The knockdown of ATF4 in GBM cell line reduced the apoptosis induced by ONC201 and the combination of ONC201 with tazemetostat or vorinostat remarkably. Therefore, induction of ISR can play an essential role in cell death of cancer cells. Apoptosis induced by ISR activation was also observed in AML cells (Ishizawa et al., 2016).

The combination of mitochondrial uncoupler niclosamide ethanolamine and dopamine receptor antagonist domperidone or TCAs induces ISR and leas to apoptosis in multiple cancer cell lines including CRC, GBM (Glioblastoma multiforme) and PDAC (Pancreatic ductal adenocarcinoma) cell lines (Hartleben et al., 2021). Even without inducing apoptosis, the ISR is induced by ONC201 in cancer cells exhibiting decreased cell proliferation (Kline et al., 2016).

The ISR contributes to drug sensitivity of cancer cells. Activation of the ISR in HER2+breast cancer contributes the sensitivity to Trastuzumab *in vivo*. Increased expression of the ISR mediator eIF2α-P predicts a better response of patients with HER2+ metastatic breast cancer to Trastuzumab therapy (Darini et al., 2019). Proteasome inhibitors are known to activate the ISR and lower expression of ISR markers thus implicating shorter progression-free survival in multiple myeloma (Obeng et al., 2006).

It was reported that ISR promotes the expression of potential target for immunotherapy (Obiedat et al., 2020). Thus, ISR may play a role in cancer immunotherapy.

On the one hand, activation of ISR plays a role in cancer therapy. On the other, Inhibition of ISR activation can increase the vulnerability of cancer cells. BCR-ABL inhibition prevents activation of ISR in K562 cell line derived from a chronic myeloid leukemia (CML) patient and makes the tumor cells more vulnerable to metabolic stress (Kato et al., 2018). Summaries of the mentioned cases and drugs can be found in the Table 1, Table 2 and Figure 3.

**TABLE 1:** The dual roles of ISR in various cancers.

Role of ISR in cancers	Cancer type
Mediator of ISR is up-regulated in anoxic tumor cells	Breast cancer Ishizawa et al. (2016)
Mediator of ISR is up-regulated in hypoxic tumor cells	Cervical cancer Hartleben et al. (2021)
Adaptation to hypoxia	Glioblastoma and colorectal cancer Darini et al. (2019)
Promotes survival of therapy-resistant hypoxic tumor cells	Glioblastoma Darini et al. (2019)
Contribute to the resistance to anoikis and promote metastasis	Fibrosarcoma Obeng et al. (2006)
ER stress is transmitted from tumor cells to myeloid cells and then facilitate tumor progression	Prostate cancer Obiedat et al. (2020)
Increase or decrease the incidence of tumor	Medulloblastoma Kato et al. (2018)
Contributes to chemoresistance	BRAF mutated melanoma Long et al. (2013)
Contributes drug sensitivity to Trastuzumab	HER2+ breast cancer Lamora et al. (2015)

**TABLE 2:** Effects of ISR compounds in the treatments of cancers.

Compounds	Effect on ISR	Effects of ISR on tumor cells	Cancer type
Gemcitabine	Induce ISR	Contributes to chemoresistance	Pancreatic cancer Palam et al. (2015)
Bortezomib	Induce ISR	Contributes drug sensitivity	Multiple myeloma Obeng et al. (2006); Narita et al. (2015)
ONC201	Induce ISR	Reduce cell-viability	Lung cancer, thyroid cancer, prostate cancer Kline et al. (2016)
ONC201	Induce ISR	Induce apoptosis	Colorectal cancer, breast cancer, glioblastoma, diffuse midline glioblastoma, AML Kline et al. (2016); Ishizawa et al. (2016); Zhang et al. (2021)
Mitochondrial uncoupler niclosamide ethanolamine + dopamine receptor antagonist domperidone or tricyclic antidepressants (TCAs)	Induce ISR	Induce apoptosis	Colorectal cancer, glioblastoma and PDAC Hartleben et al. (2021)
Nelfinavir and lopinavir	Induce ISR	Promote the expression of potential target for immunotherapy	Melanoma Obiedat et al. (2020)
BCR-ABL inhibitors	Prevent ISR activation	Enhance apoptosis	CML Kato et al. (2018)

### Manipulation of Integrated Stress Response in Cancer Therapy

The ISR takes a dual role in cell survival and cell death. Enhance or inhibition of ISR signaling *via* targeting ISR components is a promising strategy for cancer therapy (Figure 3). Among the components in ISR signaling, eIF2α is a core component and an important focused for cancer therapy.

#### Enhanced Integrated Stress Response Signaling *via* Increased eIF2α Kinase

eIF2α is a core component of the ISR, and phosphorylation of eIF2α is regulated by upstream regulators. One of approaches is to phosphorylate eIF2α by increasing eIF2α kinases upstream of eIF2α, such as GCN2, PERK, and HRI (Pakos-Zebrucka et al., 2016; Chu et al., 2021). Most of eIF2α activators are small molecules. Halofuginone and arginine deiminase are GCN2 activators (Long et al., 2013; Castilho et al., 2014). BTdCPU and ONC201 activates HRI (Kline et al., 2016; Chen et al., 2011). Bortezomib, gemcitabine, lopinavir and CCT020312 selectively activates PERK (Narita et al., 2015; Palam et al., 2015; Obeng et al., 2006; Obiedat et al., 2020; Stockwell et al., 2012). BEPP works on PKR activation (Figure 3) (Hu et al., 2009). These elF2α kinase activators have been studied in cancer therapy. For example, Halofuginone and arginine deiminase were found to inhibit tumor growth, development and metastasis either as single agents or in combination with 5-FU or radiation (Abramovitch et al., 2004; Kim et al., 2009; Cook et al., 2010; Spector et al., 2010; Lamora et al., 2015; Brin et al., 2018; Singh et al., 2019; Wang et al., 2020; Huang and Hu, 2021). Our laboratory has identified two small molecules PG3-Oc (Tian et al., 2021) and ONC201 (Kline et al., 2016; Ishizawa et al., 2016) that suppress tumor growth through increased ISR signaling. These drugs enhance ISR signaling *via* activation of eIF2α kinases, and sequentially enhance or sustain eIF2α phosphorylation.

Another approach for eIF2α phosphorylation is to prevent eIF2α dephosphorylation from eIF2α phosphatase. GADD34 (PPP1R15A) and CReP recruit phosphatase PP1 to phosphorylated-eIF2α and this results in dephosphorylation of eIF2α. Salubrinal is the first small molecule discovered to inhibit eIF2α dephosphorylation *via* both GADD34 and CReP (Boyce et al., 2005). Inhibition of GADD34 activity by Guanabenz or its derivatives results in high levels of eIF2α Phosphorylation (Tsaytler et al., 2011). Different from Guanabenz, Nelfinavir increases phosphorylation of eIF2α by downregulating CReP in addition to it effect on GADD34 (De Gassart et al., 2016). Guanabenz has been found to sensitize glioblastoma cancer cells to sunitinib in combinatorial treatment (Figure 3) (Ho et al., 2021).

#### Inhibition of Integrated Stress Response Signaling by Reduction of eIF2α Kinase

Inhibition of ISR signaling may overcome drug resistance in cancer. One of the approaches is to inhibit eIF2α kinase upstream of eIF2α. Most of these kinase inhibitors compete with ATP to block their kinase domain. SP600125 and BCR-ABL inhibitors inactivate GCN2 (Kato et al., 2018; Robert et al., 2009). Amino-pyrazolindine inhibits HRI (Rosen et al., 2009). Imidazolo-oxindole PKR inhibitor C16 specifically inhibits PKR (Jammi et al., 2003). LY-4, AMG-44, BCR-ABL inhibitors and GSK2606414 inactivate PERK (Tameire et al., 2019; Kato et al., 2018; Axten et al., 2012; Mohamed et al., 2020). They bind to the eIF2α kinase in an ATP-competitive manner, result in inhibition of kinase activity, and reduce the phosphorylation of eIF2α. Another approach is to terminate eIF2α signaling downstream of eIF2α. Small-molecule ISRIB prevents the formation of stress granules caused by eIF2α phosphorylation, thus, impairing ATF4 synthesis (Figure 3) (Sidrauski et al., 2015).

#### Targeting Integrated Stress Response in Combination of Immunotherapy

High levels of PD-L1 on the cancer cell surface allows evasion from T cell attack by binding to the PD-1 receptor on T cells. Disruption of the PD-1/PD-L1 checkpoint can result in cytotoxic T cell killing of tumors. The ISR was found to increase PD-L1 translation in human cancers. Suresh et al. (2020) The increased PD-L1 suppress anti-tumor immune responses. PERK signaling was found to suppress immune responses by increasing tumor-myeloid-derived suppressor cells (MDSC). PERK blockade transforms MDSC’s into myeloid cells that activate anti-tumor CD8+ T-cell immunity in the tumor microenvironment*.* AMG-44, a PERK inhibitor, in combination with Anti-PD-L1 showed a synergistic anti-tumor effect in B16 tumor-bearing mice model (Figure 3) (Mohamed et al., 2020). These studies suggest that PERK inhibitors enhance the antitumor efficacy of immune checkpoint inhibitors. Therefore, targeting ISR in combination with immune checkpoint is an innovational strategy for cancer therapy.

## Conclusion

The ISR is a double-edged sword with pro-survival and pro-death activities that may impact on tumor progression and response to therapy. Our approach for therapeutic targeting of cell death pathways has led us to uncover the ISR as a critical signaling component and target of drug candidates. The fact that the ISR can lead to alternative cell fates depending on cellular context suggests that greater efforts need to be directed at understanding its regulation and finding new ways for its modulation. The ISR holds promise for cancer therapy development.
